# Topological dissimilarities of hierarchical resting networks in type 2 diabetes mellitus and obesity

**DOI:** 10.1007/s10827-022-00833-9

**Published:** 2022-09-02

**Authors:** Sándor Csaba Aranyi, Zita Képes, Marianna Nagy, Gábor Opposits, Ildikó Garai, Miklós Káplár, Miklós Emri

**Affiliations:** 1 Division of Nuclear Medicine and Translational Imaging, Department of Medical Imaging, Faculty of Medicine, University of Debrecen, Debrecen, Hungary; 2 Division of Radiology and Imaging Science, Department of Medical Imaging, Faculty of Medicine, University of Debrecen, Debrecen, Hungary; 3 Translational Research Centre, ScanoMed Ltd., Debrecen, Hungary; 4 Department of Internal Medicine, Faculty of Medicine, University of Debrecen, Debrecen, Hungary

**Keywords:** Type 2 diabetes mellitus, Obesity, Effective connectivity, Dynamic causal modelling, Graph theory

## Abstract

Type 2 diabetes mellitus (T2DM) is reported to cause widespread changes in brain function, leading to cognitive impairments. Research using resting-state functional magnetic resonance imaging data already aims to understand functional changes in complex brain connectivity systems. However, no previous studies with dynamic causal modelling (DCM) tried to investigate large-scale effective connectivity in diabetes. We aimed to examine the differences in large-scale resting state networks in diabetic and obese patients using combined DCM and graph theory methodologies. With the participation of 70 subjects (43 diabetics, 27 obese), we used cross-spectra DCM to estimate connectivity between 36 regions, subdivided into seven resting networks (RSN) commonly recognized in the literature. We assessed group-wise connectivity of T2DM and obesity, as well as group differences, with parametric empirical Bayes and Bayesian model reduction techniques. We analyzed network connectivity globally, between RSNs, and regionally. We found that average connection strength was higher in T2DM globally and between RSNs, as well. On the network level, the salience network shows stronger total within-network connectivity in diabetes (8.07) than in the obese group (4.02). Regionally, we measured the most significant average decrease in the right middle temporal gyrus (-0.013 Hz) and the right inferior parietal lobule (-0.01 Hz) relative to the obese group. In comparison, connectivity increased most notably in the left anterior prefrontal cortex (0.01 Hz) and the medial dorsal thalamus (0.009 Hz). In conclusion, we find the usage of complex analysis of large-scale networks suitable for diabetes instead of focusing on specific changes in brain function.

**Supplementary Information:**

The online version contains supplementary material available at 10.1007/s10827-022-00833-9.

## Introduction

Type 2 Diabetes mellitus (T2DM) and its association with neurodegeneration and cognitive decline have received increasing attention in recent research (Moran et al., 2019; Wang et al., 2021). In the long run, diabetes leads to cognitive dysfunction and the appearance of different types of dementia (Xue et al., 2019). Several pathophysiological factors may contribute to the formation of diabetes-related cognitive impairment, such as insulinresistance, diabetes-induced inflammation, oxidative stress, and endothelial dysfunction (Galicia-Garcia et al., 2020; Infante-Garcia & Garcia-Alloza, 2019). Although the underlying mechanisms are not fully known yet, there is ongoing research to understand metabolic and cognitive alterations in the brain as a complex system. These investigations are based upon functional magnetic resonance imaging (fMRI) and positron emission tomography (PET) imaging data. Type 2 diabetic patients exhibit reduced glucose metabolism in the posterior cingulate cortex (PCC) and the precuneus (Baker et al., 2012; Piri et al., 2019). These regions are also characterized by glucose hypometabolism in Alzheimer’s disease (Cavanna & Trimble, 2006; Friedland et al., 1983), of which T2DM is generally considered as a risk factor to develop. Most fMRI studies concerning T2DM based on resting-state measurements are inferring changes in functional connectivity in resting-state networks (RSN) that is showing the coupling strength of brain regions. Chen et al. (2014) compared functional connectivity measures in the Default Mode Network (DMN) with insulin-resistance measures assessed by the homeostasis model. They found decreasing resting-state functional connectivity between PCC and the right middle temporal gyrus (rMTG) in diabetic patients with plasma glucose levels around 8–9 mmol/l and glycated hemoglobin (HbA1C) levels at 7–9%. They pointed out that aberrant DMN connectivity might be a key to understanding cognitive impairment related to T2DM. This finding is in line with the findings of Musen et al. (2012) and Zhou et al. (2010). Dai et al. (2017) found that type 2 diabetic individuals exhibit decreased cerebral blood flow in visual, cerebellum, and DMN regions after correcting for the following factors: age, hematocrit, gender, hypertension, and grey matter volume. They also pointed out that type 2 diabetes is characterized by reduced cerebral blood flow in the DMN and visual and cerebellum networks linking perfusion alterations to cognitive disability. Studies with large-scale functional connectivity analysis combined with graph theoretical methods to investigate the widespread nature of the cognitive decline in T2DM have appeared only recently (Huang et al., 2020; van Bussel et al., 2016; Xu et al., 2019). However, causal (directed) interactions between neuronal populations are rarely analyzed in T2DM; Liu et al. (2018) used Granger causality to find alterations in the connectivity of brain hubs (nodes that participate in multiple brain networks).

One of the most widely used methods to estimate directed influences over brain regions is the cross-spectral dynamic causal modelling (DCM) for resting-state fMRI data (Friston et al., 2014). This method is suitable for reasonably large-scale analysis of a network within the range of 32–64 brain regions (Razi et al., 2017). The advantage of DCM over other available effective connectivity methods is that it models the latent neural activity of each node (i.e., brain region) of the network, and estimates connection strength between them (Friston et al., 2003), rather than performing statistical comparisons among the measured fMRI data. This inversed approach is designed to analyze complex neurobiological systems and discover underlying mechanisms in the brain. To achieve this, DCM produces regional neural signals from estimated connectivity and uses the Balloon-model (Friston et al., 2000) as a hemodynamic filter to create predicted BOLD timeseries. Then, DCM iteratively improves model estimates to fit the measured fMRI data. The DCM framework also provides a way in Bayesian statistics to compare evidence of model fitness based on variational free-energy (Penny et al., 2004). Recent developments allow conducting group analysis of effective connectivity by the parametric empirical Bayes (PEB) hierarchical modeling method to estimate group-wise connectivity or group differences of the posterior parameter densities, considering the uncertainty of connections along with their estimated strength value (Friston et al., 2016).

Graph theoretical analysis for large-scale DCM results is scarce in the current literature. Still, it has been shown that averaged subject-wise connectivity can be used to measure graph characteristics (Parker Jones & Seghier, 2016). Here, we use group-level PEB models to generate representative effective connectivity matrices of both the T2DM and obese groups, which then can be transformed into weighted directed and undirected adjacency matrices.We propose a workflow that combines effective connectivity estimation performedby the DCM framework and graph theoretical analysis, where the weighted directed matrix is suitable to calculate network balance and assortativity. In contrast, the weighted undirected matrix measures average connection strength and small-world propensity parameters.

In this study, we aimed to use effective connectivity methods to find differences between resting brain connectomes of T2DM and obesity, presumably that obesity is preceding prediabetic state (Khaodhiar et al., 2009). According to previous studies on T2DM, we hypothesize that patterns of brain connectivity may distinguish people with diabetes from obese subjects. We analyzed resting-state fMRI data to estimate effective connectivity in a large-scale network of 36 regions established by Raichle (2011), subdivided into seven RSNs. We utilized the PEB method to acquire group-wise models representing T2DM and obese groups, as well as group differences. These models of group-level connectivity were used to compute graph theoreticproperties on global, RSN, and regional levels.

## Materials and methods

### Subject information

Participants for this study were selected from 96 subjects recruited from the Department of Internal Medicine of the University of Debrecen and Private General Practice from the city of Miskolc. We only analyzed the 70 subjects that took part in MRI scanning of a T1-weighted anatomical image and 10 min long resting-state fMRI data. Subject groups consist of 43 patients diagnosed with T2DM (17 female, 26 male) and 27 obese subjects (19 female, eightmale). The subject’s age was not significantly different in the two groups (mean = 50.1, sd = 8.1 for T2DM and mean = 51.7, sd = 10.1 for obesity). The following analysis fulfills the requirements of the ethical standards of the responsible national committee on human experimentation (OGYEI/2829–4/2017). Informed consent was obtained from each participating subject.

### Image acquisition

Structural and functional images were acquired at Diagnoscan, Clinical Center, University of Debrecen, using a Philips Achieva 3 T scanner. The structural 3D T1-weighted turbo field echo (TFE) images were scanned with 3.7 ms echo time (TE), 8 ms repetition time (TR), 8-degree flip angle and 0.5 × 0.5 × 1 mm^3^ voxel size. The resting-state functional images covered the whole brain, and the field-echo echo-planar imaging (FE_EPI) sequence protocol used 35 ms TE, 2300 ms TR, and a 90-degree flip angle. The in-plane resolution was 1.25 mm × 1.25 mm, and 29 interleaved axial slices were acquired with 4 mm slice thickness, leaving no gaps between slices.

### Image preprocessing

We used the nipype framework (Gorgolewski et al., 2011) to construct preprocessing pipelines. The structural preprocessing consisted of brain and tissue segmentation with Freesurfer (Fischl, 2012) version 6 and spatial normalization with ANTs (version 2.1) registration tool (Avants et al., 2008), which combines linear and non-linear transformations to achieve accurate fitting of anatomical brain images to the 2 mm isovoxel brain template (MNI152 space) created by the Montreal Neurological Institute (Grabner et al., 2006).

We preprocessed the functional images starting with motion correction using the MCFLIRT utility (Jenkinson, 2002) of the FMRIB's Software Library (FSL version 6.0). To reduce motion artifacts at later steps, we generated 24 regression variables from the six rigid-body head movement parameters (translation and rotation along three axes) as suggested by Friston et al. (1996). To reduce the impact of outlier data points on later analysis, we limited their magnitude with the 3dDespike utility of the AFNI library, version 18.3.16 (Cox & Hyde, 1997). We coregistered the functional images with the structural scanwith the FSL FLIRT tool. Then the fMRI scans were transformed into MNI152 space. At this step, we deleted the first four volumes from the time-series. After brain extraction with FSL BET, we applied the anatomical CompCor method (Behzadi et al., 2007) to compute principal components of the fMRI time-series measured in the white matter cerebrospinal fluid, which regions are not considered interesting in the analysis. We applied spatial filtering with the FSL’s application SUSAN, using a 6 mm full-width at half maximum Gaussian-kernel. Further correction of motion-related artifacts was conducted with the independent component analysis-based ICA-AROMA (Pruim et al., 2015). Then we removed the 24 motion parameters and five principal components explaining the most variance in areas denoted as noise regions from the signal in a linear regression scheme. Temporal band-pass filtering between 0.009 Hz and 0.08 Hz allowed us to analyze only the signal frequencies related to resting-state neural fluctuations. The complete preprocessing pipeline is shown on a flowchart diagram as Supplementary Information, Fig. 1.

### Region selection and coordinate adjustment

We followed the findings of Raichle (2011) to construct the basis set of brain regions. They identified 36 regions of interest (ROI) in seven RSNs: the default mode network (DMN), dorsal attention network (DAN), executive control network (CEN), salience network (SN), sensorimotor network (SMN), visual network (VN) and auditory network (AN). We provided MNI152 coordinates of each region in Table 1. To account for variability among subject-wise neuronal fluctuations, we adjusted the coordinates based on independent component analysis results. We used the GIFT toolbox (Calhoun et al., 2001) for Matlab to compute ICA on both groups of subjects. Components corresponding to synchronous fluctuations for each specific RSN were selected by the following method: first, we counted how many RSN regions were detected in each component's T statistical image with a value larger than 3. Then, we selected the component image with the highest number of matches. In cases where multiple components were found, the one with the highest mean T value across matching regions was used. Following the methods of Zhou et al. (2018), we adjusted subject-wise coordinates by looking for the peak T value on the back-reconstructed ICA images within 8 mm of the original placement.

**Table 1:** Center coordinates of the 36 regions of interest (ROI) in MNI152 space used for large-scale effective connectivity analysis

**Region name**	**Abbreviation**	**MNI X mm**	**MNI Y mm**	**MNI Z mm**
**Default mode network**				
Posterior cingulate/Precuneus	PCC	0	-52	27
Medial Prefrontal cortex	MPFC	-1	54	27
Left lateral parietal lobe	lIPL	-46	-66	30
Right lateral parietal lobe	rIPL	49	-63	33
Left inferior temporal gyrus	lITG	-61	-24	-9
Right inferior temporal gyrus	rITG	58	-24	-9
Medial dorsal thalamus	MDT	0	-12	9
Left posterior cerebellum	lPCer	-25	-81	-33
Right posterior cerebellum	rPCer	25	-81	-33
**Dorsal attention network**				
Left frontal eye field	lFEF	-29	-9	54
Right frontal eye field	rFEF	29	-9	54
Left posterior intraparietal sulcus	lPIPS	-26	-66	48
Right posterior intraparietal sulcus	rPIPS	26	-66	48
Left anterior intraparietal sulcus	lAIPS	-44	-39	45
Right anterior intraparietal sulcus	rAIPS	41	-39	45
Left middle temporal gyrus	lMTG	-50	-66	-6
Right middle temporal gyrus	rMTG	53	-63	-6
**Control executive network**				
Dorsal medial prefrontal cortex	DMPFC	0	24	46
Left anterior prefrontal cortex	lAPFC	-44	45	0
Right anterior prefrontal cortex	rAPFC	44	45	0
Left superior parietal gyrus	lSPG	-50	-51	45
Right superior parietal gyrus	rSPG	50	-51	45
**Salience network**				
Dorsal anterior cingulate cortex	DACC	0	21	36
Left anterior prefrontal cortex	lAPFC2	-35	45	30
Right anterior prefrontal cortex	rAPFC2	32	45	30
Left insula cortex	lIC	-41	3	6
Right insula cortex	rIC	41	3	6
Left lateral parietal cortex	lLPC	-62	-45	30
Right lateral parietal cortex	rLPC	62	-45	30
**Sensorimotor network**				
Left motor cortex	lM1	-39	-26	51
Right motor cortex	rM1	38	-26	48
Supplementary motor area	SMA	0	-21	48
**Visual network**				
Left primary visual cortex	lV1	-7	83	2
Right primary visual cortex	rV1	7	83	2
**Auditory network**				
Left primary auditory area	lA1	-62	-30	12
Right primary auditory area	rA1	59	-27	15

### Subject-level effective connectivity with cross-spectral DCM

Effective connectivity computations within the large-scale resting brain network were performed using dynamic causal modeling, freely available within the Statistical Parametric Mapping (SPM12 v6906) Matlab-toolbox (https://www.fil.ion.ucl.ac.uk/spm/). This method estimates causal interactions among specified brain regions in terms of normally distributed random variables of connection strength (Friston et al., 2003). The framework consists of a generative model, and an iterative parameter estimation method called variational Laplace, based on Bayesian statistics. The generative model for fMRI models neuronal interactions and hemodynamics separately to produce simulated regional blood-oxygen-level dependent (BOLD) signal from time-variant neural states, which is then compared to measurement data during parameter estimation. The specific estimation algorithm we applied for resting data (i.e.,cross-spectra DCM) uses functional connectivity in the form of predicted cross-spectra generated fromBOLD hemodynamic responses, and iteratively compares it with observed functional connectivity to fit effective connectivity parameters. In the case of large-scale networks, the advantage of comparing second-order statistics (e.g., cross spectra) instead of regional time-series is that the computationally intensive problem of estimating hidden neural states is replaced by estimating the spectral density of neuronal fluctuations. Further details of cross-spectra DCM are described in Friston et al. (2014).

For resting-state data, the following equation describes neuronal states:

$$\dot{x}\left(t\right) = A \cdot x\left(t\right) + v\left(t\right),$$

where $x\left(t\right)=[{x}_{1}\left(t\right), \dots , {x}_{n}(t)]$ is a vector of the $n$ regional neuronal states at time point $t$. The changes in neuronal states are dependent upon the states of other regions based on the $n\times n$ endogenous connectivity matrix $A$, which is the subject of the estimation procedure, and some random fluctuations $v(t)$ within the modeled brain network. Each connectivity parameter ${a}_{ij}, i\ne j$ in matrix $A$ is expressed in Hertz, as they signify the portion of neuronal excitation of region $i$ transferred to region $j$ per second. The main diagonal in $A$ represents neurologically significant self-inhibitory effects. These are log-scaling parameters (where zero corresponds to a scaling of one) that scale regional inhibition of -0.5 Hz (Friston et al., 2014).

We specified parameter priors to accommodate a fully connected network with 36 regions that formed 1296 endogenous connectivity parameters for each of the 70 subjects. Let $\theta =\left\{{\theta }^{c},{\theta }^{h}\right\}$ be the model parameters, where ${\theta }^{c}$ are the estimated coupling parameters of the matrix $A$, and ${\theta }^{h}$ are the estimated hemodynamic Balloon-model parameters (Friston et al., 2000). Then, posterior parameter estimates based on the $y$ regionally extracted BOLD data are computed based on Bayes Theorem:

$$p\left(\theta | y\right) \propto p\left(y | \theta \right) \cdot p\left(\theta \right),$$

by combining $p\left(\theta \right)$ model priors with $p\left(y | \theta \right)$ likelihood of the data. The log model evidence (LME) is approximated by negative free-energy, which maximizes the accuracy of model fit while minimizing model complexity, measured by Kullback–Leibler divergence of the prior and posterior parameter densities to avoid overfitting (Penny, 2012). Methods for parameter and hyperparameter estimation are fully described in Friston et al. (2007).

### Group-wise connectivity analysis using PEB

With recent developments in the DCM framework, we can model between-subject or higher-level connectivity with parametric empirical Bayes (PEB) (Zeidman et al., 2019). This procedure is similar to general linear modeling (GLM), as group-level connectivity data can be modeled with group effects or other confounding variables. The design matrix for PEB may contain the investigated hypotheses to be tested as well as any known (possibly) uninteresting effects (Zeidman et al., 2019).

Our analysis was conducted in two separate paths on the grouplevel. First, we estimated the main effect of estimated DCM parameters in both the T2DM and obese groups in two different PEB models. Second, we designed a between-subject PEB analysis with all subjects to reveal connectome differences between the investigated groups. For this model, the dependent variables were the subject-level estimates of the endogenous connectivity matrix, and the population baseline connectivity along with a variable to indicate groups (diabetes or obesity) were used as independent variables. The advantage of PEB over classical statistical methods for DCM parameter densities is that it correctly accounts for uncertainty in connectivity parameters (Friston et al., 2016). To assess only inter-regional interactions with the highest probability, we used Bayesian model comparison among possible models nested in the fully connected model structures. This exhaustive searching method uses Bayesian model reduction (BMR), which efficiently derives model evidence of any nested model within the base model by effectively reducing one or more parameters to its priors. Model reduction is performed until no more reduced group-level models with improved evidence can be found. Finally, an optimized model is aggregated by Bayesian model averaging from the 256 models with the highest log model evidence (Friston et al., 2016). This averaged connectivity pattern is hereinafter referred to as the BMR model, which we used for further analysis.

### Graph theory-based network analysis

#### Connectivity matrix construction

The PEB analysis combined with the BMR method produced a group-level representative effective connectivity matrix (ECM) for both groups and a matrix containing group-level connection differences (dECM). In further analysis, we used these three matrices to characterize the topological dissimilarities of the two groups’ resting-state networks.

The structure of the ECMs and the dECM are similar: they define 36 × 36 connectivity matrices, which are organized into seven RSNs. This model is essentially suitable for using the tools of graph theoretical analysis to describe the ECM topological dissimilarities and the dECM properties. However, if we examine it precisely, we see that this type of connectivity matrix can be considered as a network of networks containing signed, weighted and directed connections, and it also includes loops, as well.

Unfortunately, there is no accepted graph theoretical method for this type of hierarchical network system in brain research. To overcome this problem, we made two modifications to the ECMs: (1) removed the diagonal elements, and (2) changed the negative sign of all connection strengths to positive. This alteration produced weighted, directed connectivity matrices (WDCM) containing positive values and 0 diagonal elements. In the view of graph theory, self-connections (i.e., loops) distort degree calculation and path-length analysis used in graph metrics. Because these self-connections represent neurobiologicallyessential properties, i.e., the self-inhibitions, we analyzed them separately. The connection strength of negative values is also problematic because of their effect on the shortest path and neighbors distance calculations. Although negative connections inhibiting brain function, the energy cost of inhibitory effects may be related to the absolute value of its connection strength (Buzsáki et al., 2007). In the WDMC, the connections are not only directed but bidirected, which means that for all region pairs, there are two opposite connections. These connection-pairs are suitable for analyzing connectivity balance and generating a weighted undirected connectivity matrix (WUCM) from each WDMC by summing pairwise connection strengths.

In the case of the dECM, we did not inherit these types of matrices because its elements represent the BMR calculated connection differences. Hence, this matrix could not be considered a connectivity matrix. However, dECM is suitable for performing regional analysis by investigating increased and decreased connectivity strength relative to the two patient groups.

Using the introduced graph models, we performed three levels of graphanalysis at the global (brain), modular (RSN), and regional levels.

#### Brain-level topological characteristics

At brain-level analysis one of the most critical characteristics which can be evaluated from the WUCM is the small-world property. Small-world structure of brain networks (Bassett & Bullmore, 2006), meaning high local clustering and short average path length between any two nodes, promotes information flow in such complex systems. Hence, investigating this property became a key function for describing the topological difference between groups. For measurement of the small-worldness of the weighted network, we applied the small-world propensity (SWP) proposed by Muldoon et al. (2016) because it neglects density-dependency and keeps the essential network features such as connection strength. SWP is a quantitative metric to measure the extent to which a network shares small-world characteristics that combine the deviation of the network’s clustering coefficient (dC) and path length (dL) from a regular and a random network as well:

$$SWP=1-\sqrt{\frac{{dC}^{2}+{dL}^{2}}{2}}.$$

Methods for SWP calculations involve graph rewiring of the connectivity graph into a regular and a random graph, from which the difference of clustering coefficients and average path lengths between them can be obtained. The rewiring procedure for weighted graphs, published in Muldoon et al. (2016), is based on the formalization of small-world networks by Watts and Strogatz (1998). They placed nodes on a ring lattice with *n* vertices and *k* edges per vertex with their nearest neighbors. Then, each edge is rewired randomly with a probability of *p*. Following this concept allows us to generate graphs with anywhere between regular (*p* = *0*) and random (*p* = *1*) properties. We repeated the rewiring process 1000 times to measure SWP in both subject groups.

The other representative property of a WUCM, which is used in this analysis, is the average connection strength (S).

$$S=\sum_{i,j}{WUCM}_{i,j}.$$

This parameter can also be calculated from the diagonal values of ECMs (S_diag_). Note that in cross-spectra DCM, these values are expressed as nondimensional log scaling parameters that scale the default value of -0.5 Hz for self-inhibitory effect. This means that positive diagonal values scale up the strength of self-connections, and negative values are converted to the reduction in self-inhibition.

The WDCM matrices preserve the imbalanced strength between reciprocal effective connectivity parameters. This is useful for measuring the balance of the network. We subtracted pairwise values in the lower triangle of WDCM from the upper triangle symmetrical to the main diagonal to obtain the difference in connection strength between both directions of each region pair. Then, we performed a one-sample Student’s T-test to compare the average difference to zero, and used its negative ${\mathrm{log}}_{10}\left(p\right)$ value as a measurement of network balance.

#### RSN-level characteristics

To describe the RSNs’ topological differences, we used intra- and inter-network properties. We generated a 7 × 7 RSN-matrix (according to the seven investigated RSNs) from each graph property we calculated at the global level according to intra- and inter-network connections. The diagonal of this RSN-matrix contains the intra-RSN data, and the non-diagonal elements represent the inter-RSN properties. Another essential characteristic among RSNs is the measurement of links to occur between nodes of a similar degree is the assortativity coefficient introduced by Newman (2003) and extended to weighted graphs by Farine (2014). Using this method, the assortativity of WDCMs can be described at the brain- and RSN levels. The first one characterizes how strongly connected the regions within the RSNs are, in general. At RSN-level, we can measure the linking propensity within and between the networks on a (-1,1) standard scale.

#### Regional differences in effective connectivity

Regional differences in effective connectivity between the two groups can be measured from the dECM matrix. The differences may contain positive and negative values whether the estimated connection parameters are higher in T2DM or obesity, respectively. We calculated the average increase and decrease in connectivity that each region takes part in, separately. We reported notable connectivity differences only for regions that are below the 0.05% quantile of averaged decrease and are above the 0.95% quantile of averaged increase in connectivity among the 36 regions.

### Graphical overview of the study workflow

**Fig. 1:**
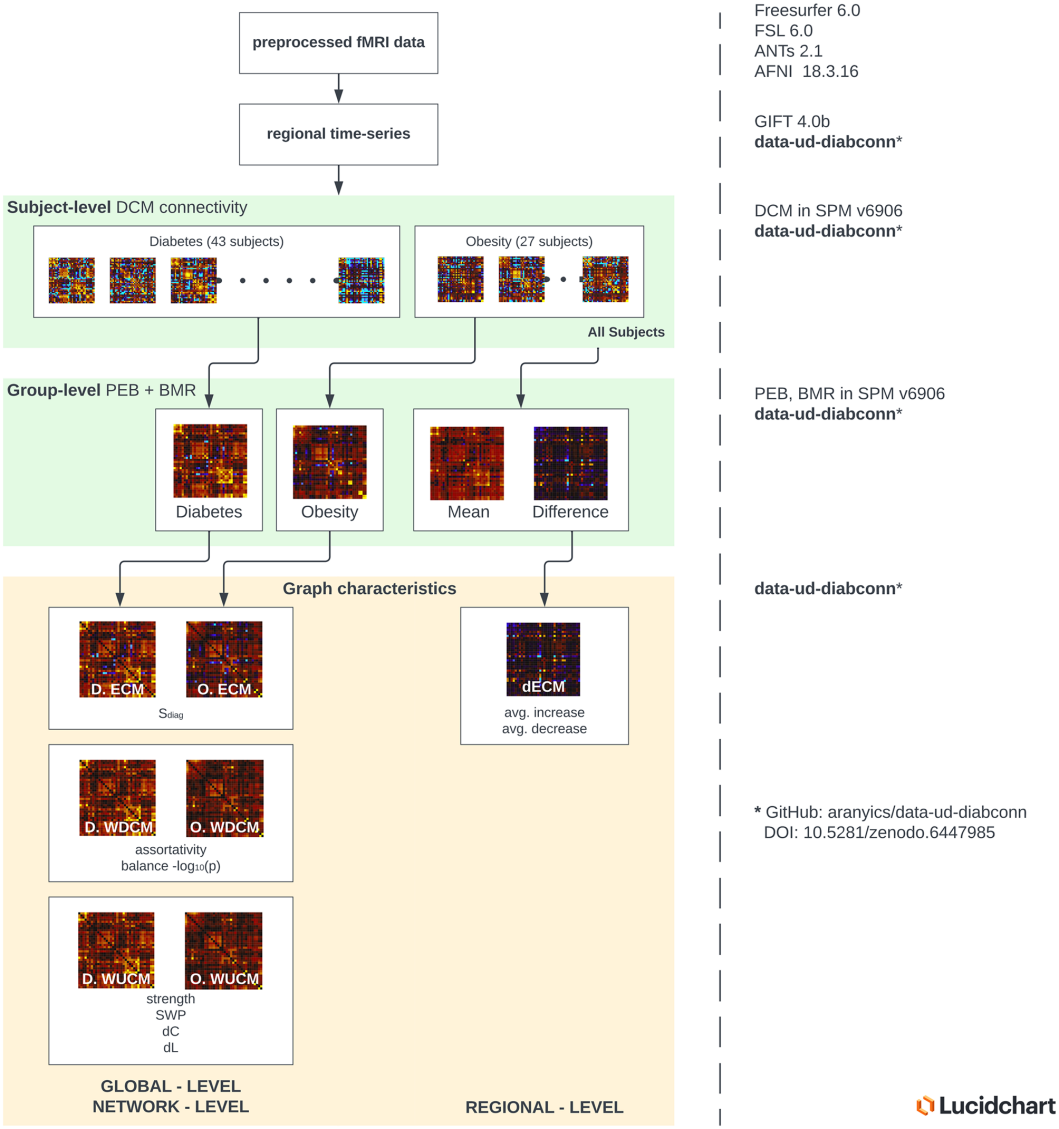
Overview of the analysis workflow for effective connectivity analysis and subsequent graph theoretical characterization. All fMRI data were previously processed through the preprocessing pipeline. We show a detailed diagram of every step implemented in our nipype pipeline in Supplementary Information, Fig. 1. Then, we extracted regional blood-oxygen-level-dependent (BOLD) time-series from the 36 regions listed in Table 1, followed by adjusting their coordinates to match group-level resting networks identified by ICA. The green boxes specify the effective connectivity analysis using methods from the DCM framework implemented in the SPM Matlab toolbox. First, subject-level DCM model parameters of fully connected networks were estimated individually for all diabetic and obese subjects. Then, we summarized group-level effective connectivity with PEB models in both groups separately, and in a third model, we also estimated group differences based on all 70 subjects. Using automatic model optimization methods based on BMR, we eliminated all PEB model parameters, which did not contribute to the final group-wise model evidence. Graph theoretical characterization of group connectivity matrices is shown in the yellow box. We used the representative matrices for diabetes and obesity to create ECMs by removing the main diagonal, which were used to compute S_diag_ inhibitory effect strength globally. From ECMs, we also derived WDCM for assortativity and balance measures and WUCM matrices to calculate the strength and small-world properties of SWP, dC, and dL on the global (whole brain) level, and within and between networks as well. Regional characteristics were only calculated from dECM of group differences to find regions with the most increase or decrease in average connectivity in T2DM patients compared to obese subjects. On the right side, we noted the used software, or the availability of research scripts at each step. The diagram was created in Lucidchart, www.lucidchart.com

We implemented a novel method to combine large-scale effective connectivity estimates on the grouplevel with graph theoretical analysis, as described in detail previously, to interpret complex neuronal interactions among resting brain networks. A diagram to summarize utilized methods can be found in Fig. 1.

## Results

In the commonly used graphanalysis, the calculations performed on the individual level offers the possibility to assess population-level graph statistics via classical hypothesis testing. However, because the DCM framework utilizes fully Bayesian methods, we had to part with classical statistical inference. Connectivity parameters in DCM describe a multivariate normal density specified by their mean, or connection strength, and the covariance (uncertainty) between parameter estimates (Friston et al., 2003). Therefore, using only the estimated strength of connections may not be sufficient for graph theoretical analysis of each subject. Our method follows the group-level workflow of PEB analysis described by Zeidman et al. (2019) instead, and omits classical statistical methods for group-level statistics.

### Description of BMR models

**Fig. 2:**
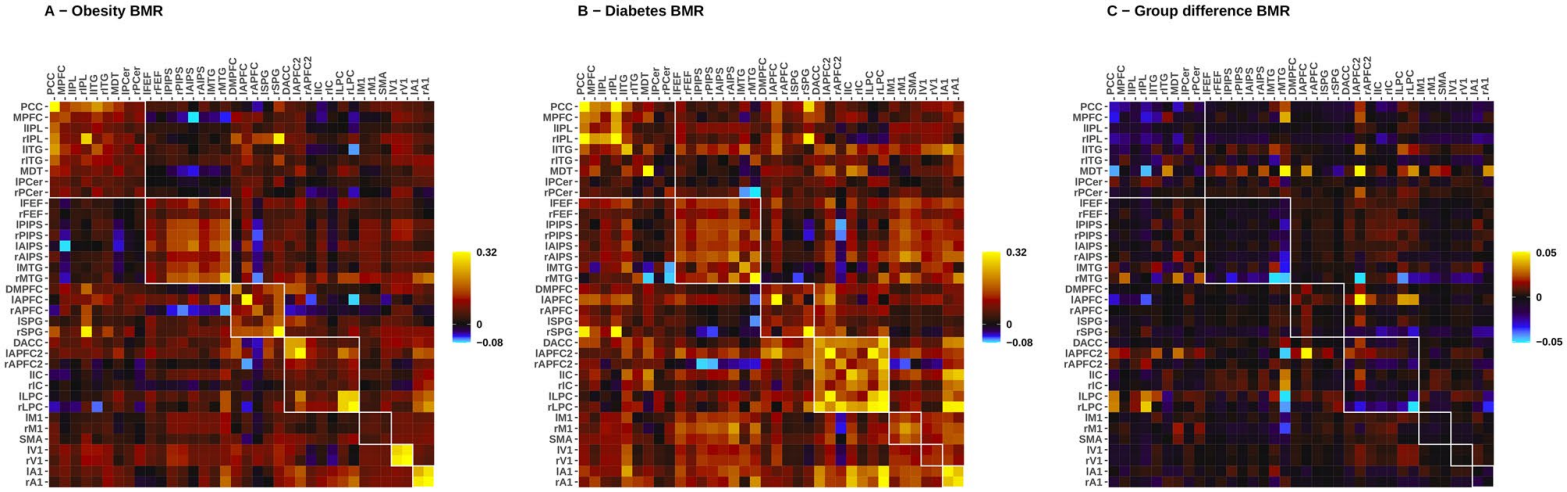
Effective connectivity matrices (ECM) representative for type 2 diabetes and obesity and their differences (dECM). Connectivity and difference matrices were acquired from the group-level BMR models. The network of 36 regions is further subdivided into seven RSNs: default mode network (DMN), dorsal attention network (DAN), control executive network (CEN), salience network (SN), sensorimotor network (SMN), visual network (VN) and auditory network (AN). Intrinsic connectivity within RSNs are displayed in the squares around the main diagonal outlined with white. Directionality is defined as regions in columns being the source and the regions in rows being the target of inter-regional interactions. For ECMs (**A** and **B**), the warm color intensity means the strength of excitation one region exerts on another expressed in Hertz (Hz), while cold colors indicate inhibitory effects. For dECM (**C**) positive values show higher connectivity in T2DM and negative values indicate a decrease in connectivity relative to obesity

The BMR models (the basis for ECMs for representative T2DM and obesity connectivity matrices and dECM connectivity differences) contain only the parameters of the second-level PEB analysis that contribute to the final model evidence. The resulting ECMs and the dECM are shown in Fig. 2. The BMR procedure removed 65 and 78 out of all 1296 parameters from the group-level representative model of T2DM and obesity, respectively. From the BMR model of group differences, 53.9% (699/1296) of all parameters were removed. Still, the large number of different connections and their relationships suggest that T2DM can be related to large-scale alterations in brain connectivity.

### Global network characteristics of the representative group-level models

#### Averaged increase in connectivity strength

Brain-level characteristics are summarized in Table 2. We found that the overall connectivity strength of regions in T2DM (6.01 Hz) is higher than in obesity (4.11 Hz), which is also found to be the case regarding diagonal (S_diag_) values between diabetes (0.25) and obesity (0.19). This means that regions are generally more interconnected in T2DM, while self-inhibition also has a higher effect in brain regions of diabetic subjects.

**Table 2:** Global graph properties for the group representative connectivity matrices

**Matrix**	**Parameter**	**Obesity**	**T2DM**	**Interpretation regarding T2DM**
ECM	S_diag_	0.188	0.246	larger self-inhibitory effect
WDCM	assortativity	0.127	0.063	weaker RSN assortativity
WDCM	balance -log_10_(p)	1.525	6.87	unbalanced T2DM connectivity
WUCM	strength	4.111	6.009	larger average strength
WUCM	SWP	0.72	0.75	small-worldness shifted to regular networks
WUCM	dC	0.28	0.17	lower clusterization coefficient
WUCM	dL	0.27	0.32	larger average path length

*ECM* effective connectivity matrix, *WDCM* weighted directed connectivity matrix, *WUCM* weighted undirected connectivity matrix, *T2DM* type 2 diabetes mellitus

#### Small-world properties

The SWP of the T2DM group's network was higher (mean = 0.75, sd = 0.006) than that of the obese group's network (mean = 0.72, sd = 0.007), which means the network for diabetes is slightly shifted towards regular networks due to larger deviation of average short path length expected from random networks (dL) in diabetes (mean = 0.32, sd = 0.009) compared to obesity (mean = 0.27, sd = 0.01). However, diabetes shows lower deviation in clusterization level (dC) than obesity (0.28 with sd = 0.011 for obese group and 0.17 with sd = 0.007 for diabetes).

#### Decreased assortativity and connectivity balance in diabetes

We used assortativity to measure how strongly regions from the same RSNs are generally connected. We found RSNs to be less assortative in T2DM (0.063) than in obesity (0.127). The non-assortative structure means that in diabetes, there are fewer similarly interconnected regions than in obesity. Regarding the bidirectional connection distribution, we found a larger imbalance in the T2DM group, showing that bidirectional connection strength difference can be significantly higher than 0 (p < 0.001).

### Differences in network-level properties between T2DM and obesity

**Fig. 3:**
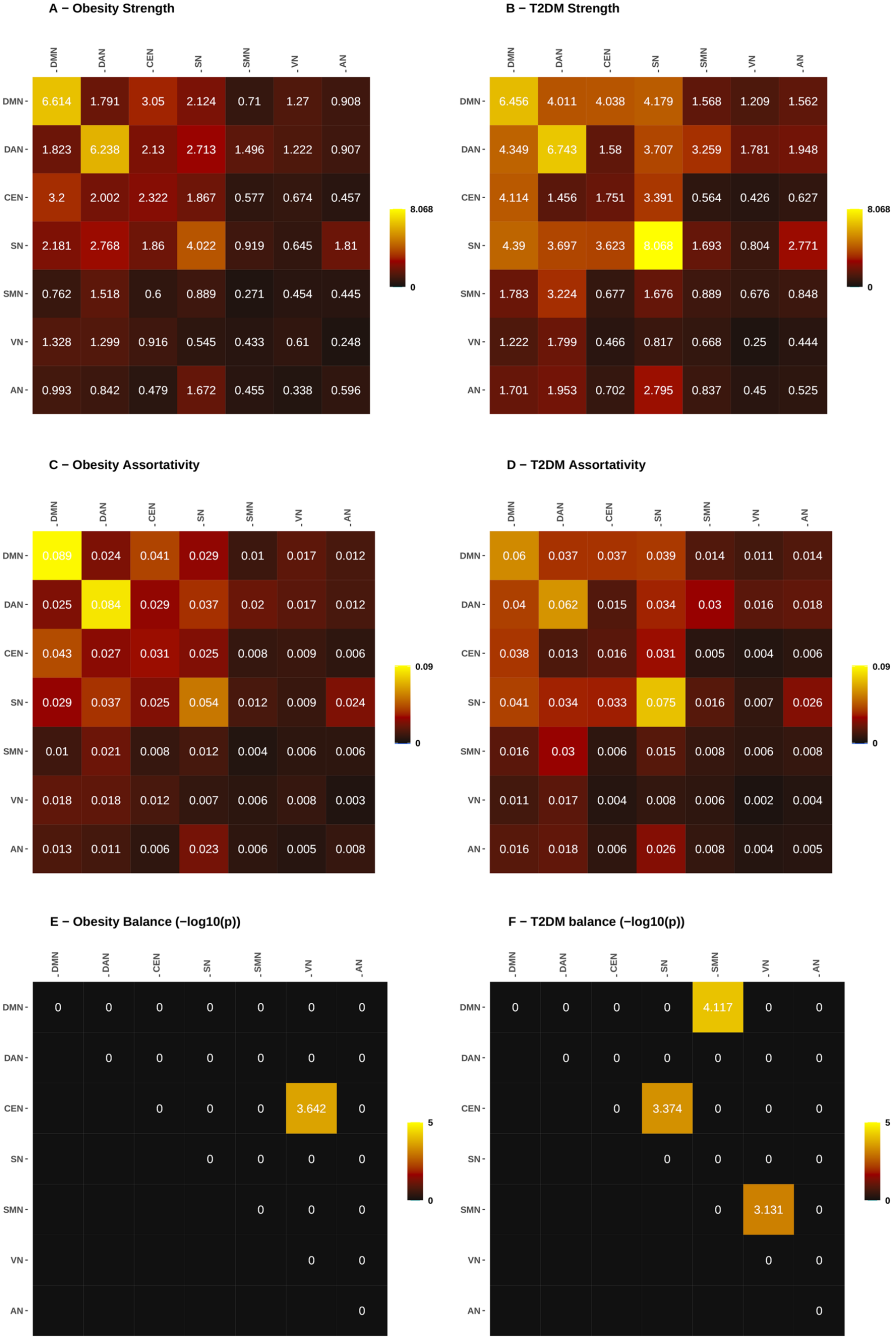
Graph properties of obese and diabetes ECMs computed on the RSN level. Diagonal elements show intra-network connections, while off-diagonal is calculated from inter-network interactions between regions of RSNs. Parameters were computed using the weighted directed connectivity matrices (WDCM). Strength, shown in the top row, describes the total connection strength between RSNs, the assortativity is in the middle row, and the bottom row shows negative log10(p) values of significantly imbalanced connectivity

**Fig. 4:**
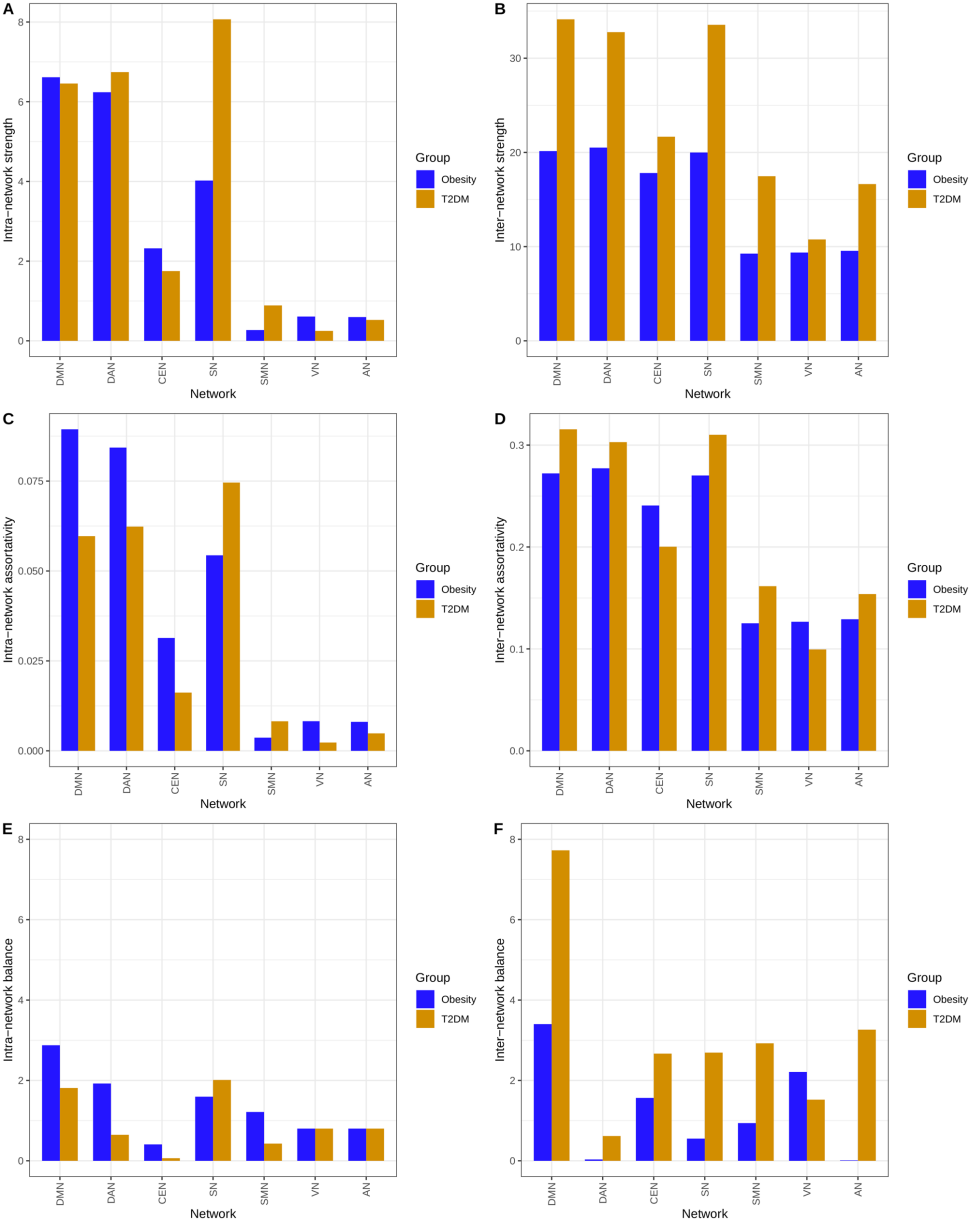
Intra- and inter-network graph properties compared between obesity and diabetes. Strength (**A**, **B**) is generally higher in diabetes, especially inter-network strength of the DMN, DAN, and SN networks and within-network connectivity in SN. Assortativity (**C**, **D**) is slightly higher in obesity within DMN and DAN while less in the SN. Imbalances (**E**, **F**) appear mostly in diabetes connectivity between RSNs, especially in the DMN

The RSN-level characteristics calculated from the WDCMs are demonstrated by the 7 × 7 RSN-matrices, where intra-network parameters are aggregated in the main diagonal, and off-diagonal values represent inter-network parameters (Fig. 3). In Table 3, we collected the average intra- and inter-network properties for illustrating the group differences. We found that both assortativity and connection strength between networks are higher in T2DM and Fig. 4 suggests that these changes originated mainly in the connectivity of DMN, DAN, and SN. Significant inter-network imbalances were found in diabetes (with negative log_10_(p) > 3), where the DMN is related to most deviations to connection balance (-log_10_(p) = 7.725).

**Table 3:** Network-level summary of applicable graph characteristics for obesity and type 2 diabetes

**Matrix**	**Parameter**	**Interaction**	**Measure**	**Obesity**	**T2DM**
WDCM	assortativity	within-network	mean	0.04	0.033
WDCM	assortativity	within-network	max	0.089	0.075
WDCM	assortativity	within-network	min	0.004	0.002
WDCM	assortativity	inter-network	mean	0.206	0.221
WDCM	assortativity	inter-network	max	0.277	0.315
WDCM	assortativity	inter-network	min	0.125	0.099
WDCM	strength	within-network	mean	2.953	3.526
WDCM	strength	within-network	max	6.614	8.068
WDCM	strength	within-network	min	0.271	0.25
WDCM	strength	inter-network	mean	15.234	23.853
WDCM	strength	inter-network	max	20.512	34.125
WDCM	strength	inter-network	min	9.259	10.761
WDCM	balance (-log_10_(p))	within-network	mean	1.374	0.938
WDCM	balance (-log_10_(p))	within-network	max	2.875	2.012
WDCM	balance (-log_10_(p))	within-network	min	0.408	0.065
WDCM	balance (-log_10_(p))	inter-network	mean	1.245	3.059
WDCM	balance (-log_10_(p))	inter-network	max	3.401	7.725
WDCM	balance (-log_10_(p))	inter-network	min	0.011	0.617

*WDCM* weighted directed connectivity matrix, *T2DM* type 2 diabetes mellitus

### Regional differences in effective connectivity

**Fig. 5:**
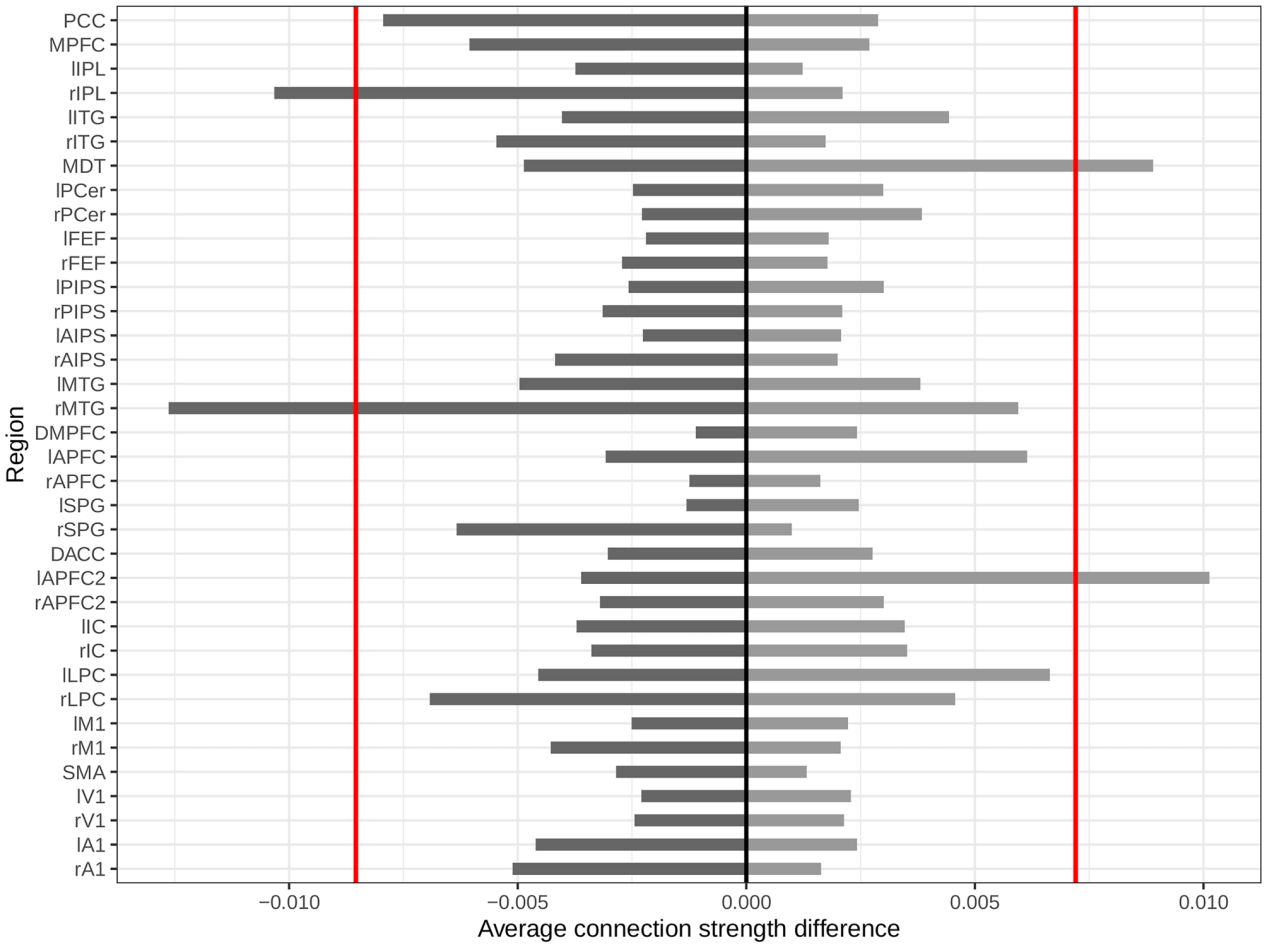
Average increase (light bars) and decrease (dark bars) in T2DM connection strength related to each region. We indicated the lower and upper 0.05% quantiles for negative and positive values, respectively, to detect outliers

We used the dECM to investigate connection differences for each region. In Fig. 5, we show the average decrease and increase in T2DM connectivity, relative to obesity, separately for each region. The most notable decrease in connectivity, exceeding the 0.05% lower quantile among all regions, is found in the right middle temporal gyrus (rMTG) with an average of 0.013 Hz decrease and in the right inferior parietal lobule (rIPL) with a 0.01 Hz decrease. On the other hand, the left anterior prefrontal cortex in the SN (lAPFC2) and the medial dorsal thalamus (MDT) have the highest increase in T2DM by 0.01 Hz and 0.009 Hz, respectively.

## Discussion

Type 2 diabetes mellitus is commonly found to cause widespread alterations in the brain. A review from Macpherson et al. (2017) summarizes the effects of T2DM on brain functions exhaustively. Most studies focused on the decreased connectivity within the DMN (Yang et al., 2016; Zhang et al., 2015; Zhou et al., 2010), especially the reduced functional connectivity of PCC and other DMN regions (Musen et al., 2012; Hoogenboom et al., 2014). Yang et al. (2016) also showed that alterations appear to be more noticeable with the progression of the disease when cognition impairment can be measured in patients. Obesity is a known risk factor for developing diabetes (Al-Goblan et al., 2014); however, the mechanisms for developing T2DM are not fully discovered. In this article, we tried to distinguish effective connectivity patterns in a large-scale brain network between obese and diabetic patients. We found that new developments in the DCM framework are helpful in analyzing effective connectivity alterations in a suitably complex system, and graph theoretical characterization of group-level PEB models may reveal significant topological dissimilarities between the connectome of subject groups.

On the global scale we observed differing graph characteristics in diabetic patients, having lower clusterization coefficient and higher characteristic path length than obese subjects. Kim et al. (2016) concluded regarding similar findings that chronic hyperglycemia may disrupt the topological integration of brain networks, which may lead to cognitive impairments.

One of our main findings was that the between-network connectivity generally increased in the majority of the networks in T2DM relative to the obese group of patients. Interestingly, overall connectivity strength increased in diabetes, especially in the SN. Based on the scope of the available literature, our results are inconclusive concerning our findings. Yang et al. (2016) reported altered, mainly decreased intra- and inter-network connectivity in type 2 diabetic individuals with or without cognitive decline. In contrast, Liu et al. (2019) also showed increased connectivity most dominantly located between SN and SMN in early T2DM patients. Based on literature data, alteration in network connectivity in diabetes could be associated with actual serum glucose, HbA1c levels and the glucose metabolic status of patients. We suppose that hyperglycaemia induced glucotoxicity could possibly contribute to the appearance of brain network alterations leading to reduced connectivity. Since our diabetic patients were well-treated and balanced regarding their glucose status, and they did not present any cognitive symptoms, hyperconnectivity could be a kind of compensatory mechanism in the early stage of disease progression. Increased inter-network connectivity strength was depicted mainly between DMN, DAN, and SN. Based on existing literature data Liu et al. (2019) also analyzed both within- and between-network connections in the mentioned networks. However, their results are partly in line with ours. They reported no significant changes regarding within-network connectivity, while increased between-network connectivity was pointed out between SMN and AN. The incoherency between the results may arise from differing methods used, or the fact that in the detailed study, people with diabetes with less than five years of disease duration were involved without any signs of cerebral small vessel diseases. In our study, we did not consider disease duration, nor did we take the effects of brain microvascular alterations into account.

Furthermore, decreased connectivity was detected between the posterior cingulate cortex (PCC) and the right middle temporal gyrus (rMTG) in both study groups. Chen et al. (2014) also had similar observations. Since both the rMTG and the PCC are brain regions related to dementia (Scheltens et al., 1992), low connectivity between them may predict the occurrence of future cognitive dysfunction in metabolic diseases. Also, decreased connectivity was detected in the rIPL in the diabetic cohort. The exact reason behind this is not yet fully elucidated. Since the region of the rIPL, similarly to that of rMTG, is a dementia-associated brain area, we speculate that altered connectivity in this region could be a promising indicator in predicting possible future cognitive decline in T2DM. However, future studies with patients with diagnosed dementia enrolled are warranted to be carried out to strengthen our hypothesis.

Network analysis might be a sensitive tool in evaluating subclinical brain alterations T2DM and obesity may cause, which could appear before cognitive clinical symptoms. Based on the relationship between network impairments and cognitive function, we hypothesize that connectivity analysis in a large-scale resting network could provide a potential biomarker for cognitive dysfunction and neurodegeneration.

One of the significant limitations of this study is the lack of a normal control group because of the difficulties of collecting data from subjects matching the patient group. Hence, we selected obesity to serve as a control condition, which is known to be a risk factor in developing insulin resistance. Furthermore, only 70 individuals out of 96 have agreed to participate in functional MRI examination up until the publication of this study.

## Conclusions

Our work aimed to investigate brain effective connectivity changes in diabetes in a large-scale model of resting networks, forming a complex system of resting-state connectivity. The advantage of using DCM to compute effective connectivity is that we can estimate causal interactions between underlying neuronal states. Using this framework and graph theory methods revealed topological differences between type 2 diabetes and obesity. We were also able to highlight regions whose interactions may elicit functional changes in the brain. We demonstrated that the usage of these types of complex analyses could aid us in further understanding the neural mechanisms of the disease or possibly finding biomarkers that may predict tendencies leading to develop type 2 diabetes.

## Supplementary Information

Below is the link to the electronic supplementary material.

Supplementary file1 (TIFF 2068 KB)

## Data Availability

The data and scripts used to produce the analysis results presented in this manuscript are available from the GitHub repository: https://github.com/aranyics/data-ud-diabconn. We have archived materials on Zenodo (https://doi.org/10.5281/zenodo.6447985).

## References

[CR1] Al-Goblan AS, Al-Alfi MA, Khan MZ (2014). Mechanism linking diabetes mellitus and obesity. Diabetes, Metabolic Syndrome and Obesity: Targets and Therapy.

[CR2] Avants B, Epstein C, Grossman M, Gee J (2008). Symmetric diffeomorphic image registration with cross-correlation: Evaluating automated labeling of elderly and neurodegenerative brain. Medical Image Analysis.

[CR3] Baker LD, Cross D, Minoshima S, Belongia D, Stennis G, Craft S (2012). Metabolism for Cognitively Normal Adults With Pre-. Archives of Neurology.

[CR4] Bassett DS, Bullmore E (2006). Small-World Brain Networks. The Neuroscientist.

[CR5] Behzadi Y, Restom K, Liau J, Liu TT (2007). A component based noise correction method (CompCor) for BOLD and perfusion based fMRI. NeuroImage.

[CR6] Buzsáki G, Kaila K, Raichle M (2007). Inhibition and Brain Work. Neuron.

[CR7] Calhoun VD, Adali T, Pearlson GD, Pekar JJ (2001). A method for making group inferences from functional MRI data using independent component analysis. Human Brain Mapping.

[CR8] Cavanna AE, Trimble MR (2006). The precuneus: A review of its functional anatomy and behavioural correlates. Brain.

[CR9] Chen Y-C, Jiao Y, Cui Y, Shang S-A, Ding J, Feng Y (2014). Aberrant Brain Functional Connectivity Related to Insulin Resistance in Type 2 Diabetes: A Resting-State fMRI Study. Diabetes Care.

[CR10] Cox RW, Hyde JS (1997). Software tools for analysis and visualization of fMRI data. NMR in Biomedicine.

[CR11] Dai W, Duan W, Alfaro FJ, Gavrieli A, Kourtelidis F, Novak V (2017). The resting perfusion pattern associates with functional decline in type 2 diabetes. Neurobiology of Aging.

[CR12] Farine DR (2014). Measuring phenotypic assortment in animal social networks: Weighted associations are more robust than binary edges. Animal Behaviour.

[CR13] Fischl B (2012). FreeSurfer. NeuroImage.

[CR14] Friedland RP, Budinger TF, Ganz E, Yano Y, Mathis CA, Koss B (1983). Regional Cerebral Metabolic Alterations in Dementia of the Alzheimer Type. Journal of Computer Assisted Tomography.

[CR15] Friston K, Mattout J, Trujillo-Barreto N, Ashburner J, Penny W (2007). Variational free energy and the Laplace approximation. NeuroImage.

[CR16] Friston KJ, Harrison L, Penny W (2003). Dynamic causal modelling. NeuroImage.

[CR17] Friston KJ, Kahan J, Biswal B, Razi A (2014). A DCM for resting state fMRI. NeuroImage.

[CR18] Friston KJ, Mechelli A, Turner R, Price CJ (2000). Nonlinear Responses in fMRI: The Balloon Model, Volterra Kernels, and Other Hemodynamics. NeuroImage.

[CR19] Friston KJ, Litvak V, Oswal A, Razi A, Stephan KE, van Wijk BCM (2016). Bayesian model reduction and empirical Bayes for group (DCM) studies. NeuroImage.

[CR20] Friston KJ, Williams S, Howard R, Frackowiak RSJ, Turner R (1996). Movement-Related effects in fMRI time-series. Magnetic Resonance in Medicine.

[CR21] Galicia-Garcia U, Benito-Vicente A, Jebari S, Larrea-Sebal A, Siddiqi H, Uribe KB (2020). Pathophysiology of Type 2 Diabetes Mellitus. International Journal of Molecular Sciences.

[CR22] Gorgolewski K, Burns CD, Madison C, Clark D, Halchenko YO, Waskom ML, Ghosh SS (2011). Nipype: A Flexible, Lightweight and Extensible Neuroimaging Data Processing Framework in Python. Frontiers in Neuroinformatics.

[CR23] Grabner G, Janke AL, Budge MM, Smith D, Pruessner J, Collins DL (2006). Symmetric Atlasing and Model Based Segmentation: An Application to the Hippocampus in Older Adults. Medical Image Computing and Computer-Assisted Intervention – MICCAI 2006. MICCAI 2006. Lecture Notes in Computer Science.

[CR24] Hoogenboom, W. S., Marder, T. J., Flores, V. L., Huisman, S., Eaton, H. P., Schneiderman, J. S., Bolo, N. R., Simonson, D. C., Jacobson, A. M., Kubicki, M., Shenton, M. E., & Musen, G. (2014). Cerebral white matter integrity and resting-state functional connectivity in middle-aged patients with type 2 diabetes. *Diabetes*, *63*(2), 728–738. 10.2337/db13-1219 10.2337/db13-1219 PMC3900542 24203723

[CR25] Huang X, Tong Y, Qi C-X, Dan H-D, Deng Q-Q, Shen Y (2020). Large-Scale Neuronal Network Dysfunction in Diabetic Retinopathy. Neural Plasticity.

[CR26] Infante-Garcia C, Garcia-Alloza M (2019). Review of the Effect of Natural Compounds and Extracts on Neurodegeneration in Animal Models of Diabetes Mellitus. International Journal of Molecular Sciences.

[CR27] Jenkinson M (2002). Improved Optimization for the Robust and Accurate Linear Registration and Motion Correction of Brain Images. NeuroImage.

[CR28] Khaodhiar L, Cummings S, Apovian CM (2009). Treating diabetes and prediabetes by focusing on obesity management. Current Diabetes Reports.

[CR29] Kim D-J, Yu JH, Shin M-S, Shin Y-W, Kim M-S (2016). Hyperglycemia Reduces Efficiency of Brain Networks in Subjects with Type 2 Diabetes. PLoS ONE.

[CR30] Liu D, Duan S, Zhou C, Wei P, Chen L, Yin X (2018). Altered Brain Functional Hubs and Connectivity in Type 2 Diabetes Mellitus Patients: A Resting-State fMRI Study. Frontiers in Aging Neuroscience.

[CR31] Liu Q, Zeng X, Jiang X-M, Zhou Z, Hu X (2019). Altered Brain Functional Hubs and Connectivity Underlie Persistent Somatoform Pain Disorder. Frontiers in Neuroscience.

[CR32] Macpherson H, Formica M, Harris E, Daly RM (2017). Brain functional alterations in Type 2 Diabetes – A systematic review of fMRI studies. Frontiers in Neuroendocrinology.

[CR33] Moran C, Beare R, Wang W, Callisaya M, Srikanth V, Weiner M (2019). Type 2 diabetes mellitus, brain atrophy, and cognitive decline. Neurology.

[CR34] Muldoon SF, Bridgeford EW, Bassett DS (2016). Small-World Propensity and Weighted Brain Networks. Scientific Reports.

[CR35] Musen G, Jacobson AM, Bolo NR, Simonson DC, Shenton ME, McCartney RL (2012). Resting-State Brain Functional Connectivity Is Altered in Type 2 Diabetes. Diabetes.

[CR36] Newman MEJ (2003). Mixing patterns in networks. Physical Review E.

[CR37] Parker Jones O, Seghier M (2016). Graph theoretic analysis on large DCM models. Frontiers in Neuroscience.

[CR38] Penny WD (2012). Comparing Dynamic Causal Models using AIC, BIC and Free Energy. NeuroImage.

[CR39] Penny WD, Stephan KE, Mechelli A, Friston KJ (2004). Comparing dynamic causal models. NeuroImage.

[CR40] Piri R, Naghavi-Behzad M, Gerke O, Høilund-Carlsen PF, Vafaee MS (2019). Investigations of possible links between Alzheimer’s disease and type 2 diabetes mellitus by positron emission tomography: A systematic review. Clinical and Translational Imaging.

[CR41] Pruim RHR, Mennes M, van Rooij D, Llera A, Buitelaar JK, Beckmann CF (2015). ICA-AROMA: A robust ICA-based strategy for removing motion artifacts from fMRI data. NeuroImage.

[CR42] Raichle ME (2011). The Restless Brain. Brain Connectivity.

[CR43] Razi A, Seghier ML, Zhou Y, McColgan P, Zeidman P, Park H-J (2017). Large-scale DCMs for resting-state fMRI. Network Neuroscience.

[CR44] Scheltens P, Leys D, Barkhof F, Huglo D, Weinstein HC, Vermersch P (1992). Atrophy of medial temporal lobes on MRI in “probable” Alzheimer’s disease and normal ageing: Diagnostic value and neuropsychological correlates. Journal of Neurology, Neurosurgery & Psychiatry.

[CR45] van Bussel FCG, Backes WH, van Veenendaal TM, Hofman PAM, van Boxtel MPJ, Schram MT (2016). Functional Brain Networks Are Altered in Type 2 Diabetes and Prediabetes: Signs for Compensation of Cognitive Decrements? The Maastricht Study. Diabetes.

[CR46] Wang Y, Sun L, He G, Gang X, Zhao X, Wang G, Ning G (2021). Cerebral perfusion alterations in type 2 diabetes mellitus – a systematic review. Frontiers in Neuroendocrinology.

[CR47] Watts DJ, Strogatz SH (1998). Collective dynamics of ‘small-world’ networks. Nature.

[CR48] Xu J, Chen F, Liu T, Wang T, Zhang J, Yuan H, Wang M (2019). Brain Functional Networks in Type 2 Diabetes Mellitus Patients: A Resting-State Functional MRI Study. Frontiers in Neuroscience.

[CR49] Xue M, Xu W, Ou YN, Cao XP, Tan MS, Tan L, Yu JT (2019). Diabetes mellitus and risks of cognitive impairment and dementia: A systematic review and meta-analysis of 144 prospective studies. Ageing Research Reviews.

[CR50] Yang S-Q, Xu Z-P, Xiong Y, Zhan Y-F, Guo L-Y, Zhang S (2016). Altered Intranetwork and Internetwork Functional Connectivity in Type 2 Diabetes Mellitus With and Without Cognitive Impairment. Scientific Reports.

[CR51] Zeidman P, Jafarian A, Seghier ML, Litvak V, Cagnan H, Price CJ, Friston KJ (2019). A guide to group effective connectivity analysis, part 2: Second level analysis with PEB. NeuroImage.

[CR52] Zhang H, Hao Y, Manor B, Novak P, Milberg W, Zhang J (2015). Intranasal Insulin Enhanced Resting-State Functional Connectivity of Hippocampal Regions in Type 2 Diabetes. Diabetes.

[CR53] Zhou H, Lu W, Shi Y, Bai F, Chang J, Yuan Y (2010). Impairments in cognition and resting-state connectivity of the hippocampus in elderly subjects with type 2 diabetes. Neuroscience Letters.

[CR54] Zhou Y, Friston KJ, Zeidman P, Chen J, Li S, Razi A (2018). The Hierarchical Organization of the Default, Dorsal Attention and Salience Networks in Adolescents and Young Adults. Cerebral Cortex.

