# Supplementary figures and images for: Topological dissimilarities of hierarchical resting networks in type 2 diabetes mellitus and obesity
Source: J Comput Neurosci. 2022 Sep 2;51(1):71–86. doi: 10.1007/s10827-022-00833-9 (PMC9840595; doi:10.1007/s10827-022-00833-9)

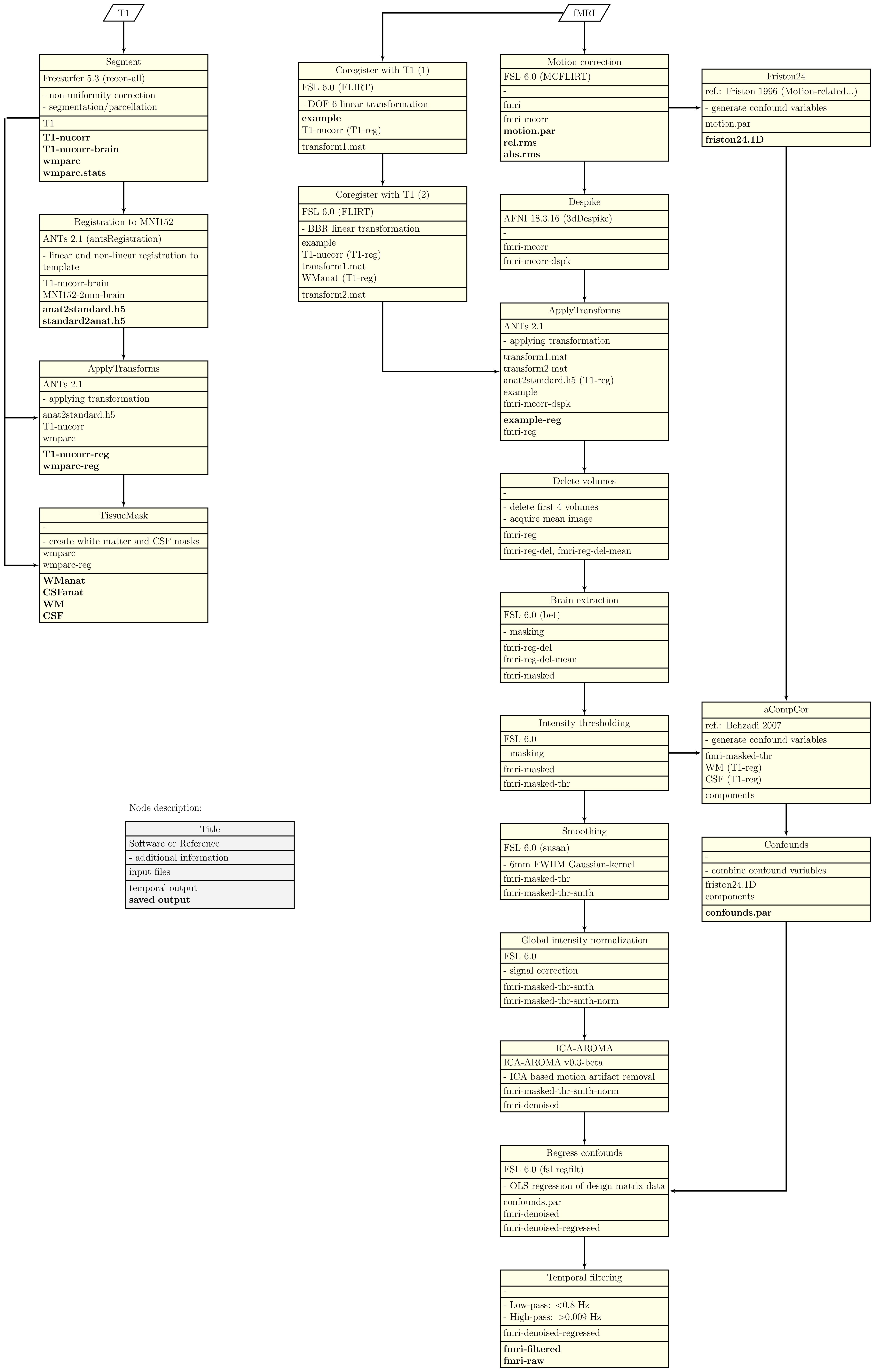

Supplement: Supplementary file 1 — Supplementary file1 (TIFF 2068 KB) [file 10827_2022_833_MOESM1_ESM.tiff]
